# Science knowledge and trust in medicine affect individuals’ behavior in pandemic crises

**DOI:** 10.1007/s10212-021-00529-1

**Published:** 2021-04-14

**Authors:** Michael Sailer, Matthias Stadler, Elouise Botes, Frank Fischer, Samuel Greiff

**Affiliations:** 1 Department of Psychology, Ludwig-Maximilians-Universität München, Munich, Germany; 2 University of Luxembourg, Esch-Sur-Alzette, Luxembourg

**Keywords:** COVID-19, SARS-CoV-2, Coronavirus, Science knowledge, Trust in medicine, Individuals' behavior

## Abstract

In pandemic crises such as the COVID-19 pandemic, individuals’ behavior has a strong impact on epidemiological processes. Compliance with prevention guidelines, such as social distancing, is critical to avoid further spreading an infectious disease or to slow down its spread. However, some individuals also or instead engage in panic behavior, such as hoarding. We investigate how education prepares individuals to respond adequately by modelling the path from seeking information about COVID-19 to eventual behavior. Based on a sample of *N* = 1182 adult Americans, gathered at the pandemic’s onset (March 2020), we conclude that science knowledge helps individuals convert information into coronavirus knowledge. This knowledge then helps individuals avoid panic behavior. Individuals lacking coronavirus knowledge and science knowledge still comply with prevention guidelines when they have a general trust in medicine. Individuals lacking knowledge still follow prevention guidelines when they trust in medicine. Facilitating science knowledge and trust in science through education and targeted public health messaging are likely to be of fundamental importance for bringing crises such as the COVID-19 pandemic under control.

## Introduction

In public health crises such as the COVID-19 pandemic, individuals’ behavior has a strong impact on epidemiological processes during critical stages of the outbreak (Anderson et al. 2020; Bish and Michie 2010). It is critical for individuals to engage in preventive behavior at the outbreak of a pandemic, such as social distancing (Glass et al. 2006) or refraining from going to work with symptoms, in order to slow down or avoid the spread of infectious diseases. In addition, panic behavior, such as hoarding or dangerous self-medication (Mowbray 2020), must be avoided to ensure public order and prevent individuals from harming themselves.

Knowledge about a crisis such as the COVID-19 pandemic has been found to be among the most critical predictors of whether people will respond adequately (Clements 2020). Therefore, education about the disease and the necessity of preventive measures are pivotal for public compliance. However, individual differences in general scientific knowledge limits people’s ability to fully grasp the complex information necessary to develop an understanding of an emerging disease that is not even fully understood by experts. Moreover, a personal rejection of science may influence people to not follow recommendations for addressing science-based problems (Solomon 1993).In this study, we investigate how different types of scientific knowledge and trust in medicine can help individuals comply with preventive measures and prevent them from engaging in panic behavior during the onset of pandemic crises.

### Individual behavior in the COVID-19 pandemic crisis

A new type of coronavirus called SARS-CoV-2 emerged in December 2019 in Wuhan, Hubei Province, China (Zhou et al. 2020). COVID-19 has since rapidly emerged as a global health threat, with some calling it a once-in-a-century pandemic (Gates 2020; Lai et al. 2020). To date, no vaccine is available to protect individuals from COVID-19 and no drugs can cure COVID-19 (Anderson et al. 2020). In light of this situation, individual behavior will be crucial to controlling the spread of COVID­19 (Anderson et al. 2020; Chen et al. 2020; Larson and Nigmatulina 2009), as it has a strong impact on epidemiological processes during critical stages of the outbreak (Anderson et al. 2020).

Unprecedented public health measures such as quarantines, social distancing, community containment, and isolation of infected populations have been initiated globally to contain the further spread of the disease (Anderson et al. 2020; Wilder-Smith and Freedman 2020). However, such measures are ethically challenging, involve major restrictions on individual freedom, and require high levels of individual commitment (Wilder-Smith and Freedman 2020). Thus, the success of these measures is highly dependent on the behavior of individuals. Engaging in early self-isolation, seeking medical advice remotely unless symptoms are severe, and social distancing are critical for controlling the spread of COVID­19 (Anderson et al. 2020). The vast majority of individuals seem to be committed to comply with such preventive measures (Glass et al. 2006). We define compliance with preventive measures as the willingness of individuals to follow evidence-based guidelines, e.g. by the World Health Organization (WHO) or the Centers for Disease Control and Prevention (CDC). However, there are reports of individuals also or instead engaging in panic behavior, such as hoarding (Mahase 2020). Panic behavior overwhelmingly results from fear and can be defined as behavior that evidence-based guidelines classify as unreasonable or unnecessary (e.g., panic buying), or as harmful (e.g. self-medication; see Garfin et al. 2020; Mowbray 2020). Panic buying and hoarding behavior can be dangerous to public order as it can lead to global shortages and price gouging for important necessities, such as toilet paper or hand sanitizer (Garfin et al. 2020). In addition, other types of panic behavior, such as dangerous self-medication, pose a personal risk for the individuals who engage in them (Mowbray 2020). We are aware that the adequacy of behaviors is indefinite and can change over time based on available evidences. We assume that an individual can simultaneously both follow preventive guidelines and engage in panic behavior. Thus, individual behavioral responses to the COVID-19 pandemic might be best explained by two different models that, to our knowledge, have not before been empirically investigated.

Understanding factors that influence how individuals behave during the COVID-19 outbreak is crucial both for controlling the epidemic’s spread and severity (Bish and Michie 2010) and for maintaining public order. Two such factors are individual information seeking that is paying attention to and searching for information regarding COVID-19 (see Feldman et al. 2012), and (science) education. Individuals depend on the media to receive accurate and up-to-date information on what they and the health system need to do without causing panic (Garfin et al. 2020). The central question of this article is how individuals’ information seeking leads to different types of behavior, namely compliance with official guidelines and panic behavior. There are two major ways in which information can modify behavior. Firstly, should the source of information be judged as highly trustworthy, advice might be followed without first being evaluated (Chinn and Duncan 2018). Secondly, information might be critically evaluated based on one’s own knowledge. Applying this line of reasoning on the COVID-19 pandemic, we assume both a direct relation between individuals’ information seeking and behavior and an indirect relation mediated by coronavirus knowledge. In addition, general science knowledge should help in evaluating information, whereas trust in medicine should play a crucial role in whether recommendations are actually followed.

### The role of science knowledge

Actively seeking out information can positively influence the knowledge individuals acquire on a topic (Eveland 2001). This is also true for the coronavirus, where the knowledge individuals acquire can lead to different types of behavior or behavioral intentions during pandemic crises (Ho et al. 2013). Specifically, knowledge can help foster the intention to comply with preventive measures (Ho et al. 2013) and avoid panic behavior. The extent to which specific knowledge is the result of actively seeking out information and appropriately understanding this information, is dependent on how this information is elaborated in memory (Eveland 2001; Ho et al. 2013) based on individuals’ existing knowledge networks. More specifically, the interpretation and adequate use of information about the coronavirus depends on individuals’ ability to engage in science-based reasoning (Fischer et al. 2014) and requires scientific literacy (Benjamin et al. 2017). Thus, general science knowledge (McPhetres and Zuckerman 2018) should facilitate the acquisition of coronavirus knowledge as a specific form of knowledge. Such knowledge is crucial and has been shown to be of significant value for performance in scientific reasoning tasks (Chinn and Duncan 2018). Applying science knowledge to evaluate and interpret evidence is known as first-order scientific reasoning and can directly enable individuals to successfully complete reasoning tasks by making knowledge-based decisions (Chinn and Duncan 2018). This form of scientific reasoning, which leads to specific knowledge relevant for the current pandemic situation, might in turn influence individuals’ behavior.

### The role of trust

In addition to first-order scientific reasoning, which involves reasoning with evidence, individuals can also engage in second-order scientific reasoning. Second-order scientific reasoning refers to reasoning regarding which experts or sources to trust on the science topic in question (Chinn and Duncan 2018). Trust in science and, in the case of a pandemic crisis, trust in medicine in particular might play a major role in reasoning processes and related behavior. Trust in science in general has been found to be positively related to compliance with COVID-19 prevention guidelines (Plohl and Musil 2020). Trust in medicine in particular has been found to positively affect individuals’ willingness to adopt recommended behavior in pandemic crises (Siegrist and Zingg 2014). Situations such as pandemic crises are usually highly complex and thus making good decisions is difficult for individuals, particularly when they lack knowledge and encounter a multitude of information that is difficult to process (Siegrist et al. 2000; Siegrist and Zingg 2014). Trust can help individuals rely on others in their decision-making. Furthermore, trust can be important in helping individuals select people or organizations whose recommendations they should follow (Siegrist and Zingg 2014).

### The present study

In this study, we investigate how individuals’ active information seeking leads to compliance with preventive measures and panic behavior related to the coronavirus. Furthermore, we examine the interaction between knowledge and trust, which might play a crucial role in individuals’ engagement in different types of behavior.

We collected data in the United Stated of America (USA) during the COVID-19 pandemic’s onset between March 13^th^ and 15^th^ 2020. Important context information about the situation globally and in the USA at that time are the following: On March 11^th^ the WHO declared the outbreak to be a pandemic. The WHO generally encouraged social distancing and specifically recommended that people with mild respiratory symptoms isolate themselves (Cucinotta and Vanelli 2020). Furthermore, the WHO also warned of a disruption in the global supply of personal protective equipment due to rising demand, hoarding, and misuse (Mahase 2020).

On March 11^th^ 2020, the USA surpassed 1,100 confirmed coronavirus cases (Johns Hopkins University Medicine 2020). Furthermore, on the same day, the president of the USA addressed the nation and suspended all travel from Europe starting March 13^th^ 2020. Travel from China and South Korea had already been restricted. He highlighted the importance of practicing good hygiene, i.e. washing hands, cleaning often-used surfaces, covering face and mouth when sneezing or coughing, and staying at home when feeling sick (The White House 2020).

Against this background, the following research questions will be investigated:

- RQ1: How does information seeking relate to compliance with preventive measures behavior in a pandemic crisis and to what extent is this relationship dependent on specific knowledge about the coronavirus, science knowledge, and trust in medicine?
- RQ2: How does information seeking relate to panic behavior in a pandemic crisis and to what extent is this relationship dependent on specific knowledge about the coronavirus, science knowledge, and trust in medicine?

## Methods

### Sample and procedure

Data was collected between March 13^th^ and 15^th^ 2020 through an online questionnaire hosted by SoSci Survey (https://www.soscisurvey.de/). The questionnaire was distributed through Prolific, a paid research participant recruitment site (www.prolific.co). The survey link was sent via Prolific to a sample of citizens of the USA that was stratified for age and gender. Participants were compensated for their time via Prolific. Data collection procedures received the necessary ethical approval from the Ethics Review Panel of the University of Luxembourg (ERP 20-015 PANIC).

A total of 1,217 participants completed the survey; however, 31 participants had a substantial amount of missing data and were thus excluded from the dataset. An additional 4 participants indicated that they had tested positive for coronavirus and were excluded from the dataset. The final sample consisted of *N* = 1182 citizens of the USA.

The average age of the sample was 45.6 years (SD = 15.72), with a minimum age of 20 years old and a maximum of 83. The sample further consisted of 50.4% female and 48.6% male participants, with 1% not indicating a gender. The majority of the sample was White (76.4%), followed by African American/Black (11.9%), and Asian (5.4%). Regarding education, the largest number of participants reported having obtained a Bachelor’s degree (35%), followed by some tertiary education without obtaining a degree (22.8%), Master’s degree (13.2%), and Associate’s degree (11.8%). Only very few participants (5.6%) had obtained a professional degree (e.g., MD) or a doctorate (e.g., PhD).

The complete data as well as all measures used can be found in the open science framework repository (https://osf.io/dkv2s/).

### Measures

All items, including scales not used in this study, can be found on an open science repository (https://osf.io/dkv2s/).

#### Information seeking

Information seeking was assessed with three items adapted from Feldman et al. (2012). Participants rated their agreement with statements such as “I pay attention to information about Coronavirus” on a scale from 1 (strongly disagree) to 5 (strongly agree). All scores were aggregated into one mean score (*ω* = 0.86).

#### Compliance with preventive measures

Compliance with preventive measures was assessed by asking participants to rate their agreement with seven statements such as “I am planning to/have already started avoiding crowded spaces” on a scale from 1 (strongly disagree) to 5 (strongly agree). All scores were aggregated into one mean score (*ω* = 0.86).

#### Panic behavior

Panic behavior was assessed by asking participants to rate their agreement with seven statements such as “I am planning to/have already bought more toilet paper than usual” on a scale from 1 (strongly disagree) to 5 (strongly agree). All scores were aggregated into one mean score (*ω* = 0.78).

The set of items for both types of behavior are based on the evidence available on March 13, 2020, at 11:15 am (GMT + 1).

#### Coronavirus knowledge

Knowledge about the coronavirus refers to knowledge about both SARS-CoV-2 and COVID-19 and was assessed using 16 questions covering “Symptoms,” “Transmission and Course,” and “Treatments and Prevention” of the coronavirus. Each question consisted of four statements, only one of which was correct (e.g., “Most Coronavirus patients will suffer a fever, cough, and some breathing difficulties”), while the others were incorrect (e.g., “Coronavirus is characterized by swelling and tenderness in your lymph nodes”). All questions were developed by the authors and based on existing knowledge on March 13, 2020, at 11:15 am (GMT + 1). Answers were scored as either correct or incorrect, with the percentage of correct answers representing the participants’ final score. As suggested by Stadler et al. (2021), we computed variance inflation factors (VIF) for all items to avoid having redundant items representing our formative knowledge construct. The maximum VIF was VIF_max_ = 1.45, which is below the recommended cut-off of 3.3.

#### Science knowledge

Science knowledge was assessed using a scale by McPhetres and Zuckerman (2018). Participants indicated whether they thought each of 12 statements were true (e.g., “Light travels faster than sound”) or false (e.g., “Antibiotics kill viruses as well as bacteria”). The statements covered a wide range of scientific topics, including biology, physics, and chemistry. Answers were scored as either correct or incorrect, with the percentage of correct answers representing the final score. There were no redundant items (VIF_max_ = 1.27).

#### Trust in medicine

Trust in medicine was assessed using the Trust in the Medical Profession Scale (Dugan et al. 2005). Participants rated their agreement with five statements such as “I completely trust doctors’ decisions about which medical treatments are best” on a scale from 1 (strongly disagree) to 5 (strongly agree). All scores were aggregated into one mean score (ω = 0.86).

### Statistical analysis

**Fig. 1:**
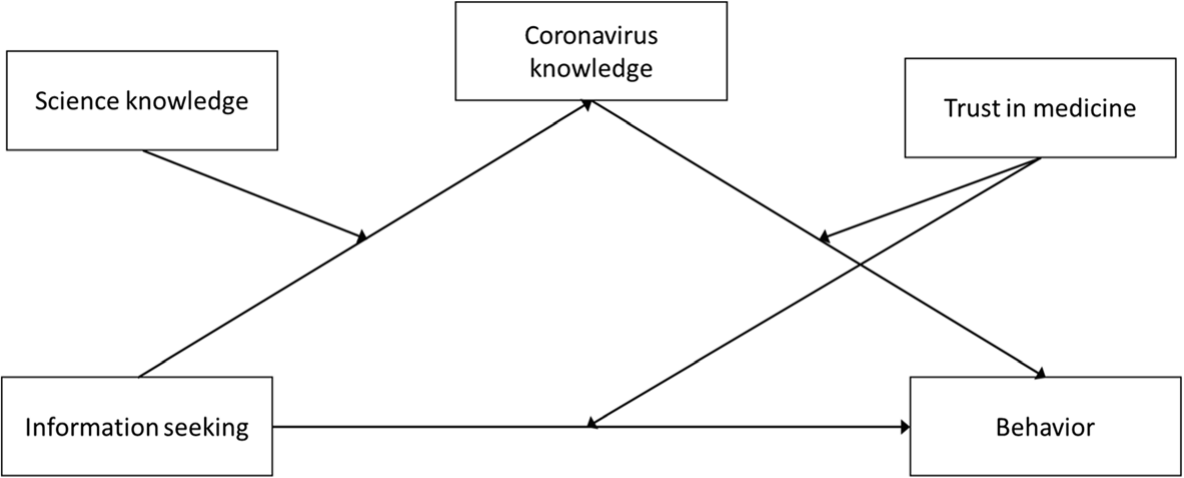
Graphical illustration of the moderated mediations. The behavior in the moderated mediation models was either compliance with preventive measures (Model 1) or panic behavior (Model 2)

We defined two moderated mediation models (Hayes 2013) with knowledge about the coronavirus mediating the relation between information seeking and compliance with preventive measures (Model 1) or panic behavior (Model 2). For both models, we defined science knowledge as a moderator of the relation between information seeking and knowledge about the coronavirus. That is, the relation between information seeking and knowledge about the coronavirus depends on the level of science knowledge. Furthermore, we defined trust in medicine as a moderator of both the relation between information seeking and behavior as well as the relation between knowledge about the coronavirus and behavior. Figure 1 graphically depicts the moderated mediations. All analyses were conducted using R 3.5.2 (R Core Team 2018). The complete code including all packages used can be found in the open science framework repository https://osf.io/dkv2s/.

## Results

### Descriptive statistics

Table 1 displays descriptive statistics for all variables. The means of both coronavirus knowledge and science knowledge were high (values ranging from 0 to 1, representing the percentage of correct answers). Most participants reported high levels of information seeking and compliance with preventive measures, while mostly avoiding panic behavior (values ranging from 1 to 5, with higher values indicating higher levels of the corresponding attribute). Trust in medicine was relatively low. All assumed bivariate correlations were substantial and in the expected direction. Interestingly, compliance with preventive measures correlated slightly positive with panic behavior (*r* = 0.27), indicating that participants who complied with preventive measures were also more likely to show panic behavior. There were no substantial relations between participants’ age and any of the variables (all *r* < 0.2), nor were there any differences between genders (all *d* < 0.2).

**Table 1:** Means, standard deviations, and correlations for all variables; *M* and SD represent mean and standard deviation, respectively; * indicates *p* < .05; ** indicates *p* < .01

Variable	*M*	*SD*	1	2	3	4	5
1. Information seeking	4.14	0.82					
2. Compliance with preventive measures	3.93	0.81	.47**				
3. Panic behavior	1.79	0.71	.10**	.27**			
4. Trust in medicine	2.74	1.07	.09**	.04	−.14**		
5. Coronavirus knowledge	0.78	0.14	.31**	.13**	−.30**	.08**	
6. Science knowledge	0.80	0.17	.05	−.10**	−.34**	.07*	.43**

### Compliance with preventive measures (Research Question 1)

In Model 1, we investigated how information seeking relates to compliance with preventive measures in a pandemic crisis and to what extent this relationship depends on specific knowledge about the coronavirus, science knowledge, and trust in medicine. Information seeking (*β* = 0.22; *p* < 0.001), science knowledge (*β* = 0.35; *p* < 0.001), and the interaction between them (*β* = 0.11; *p* = 0.002) were all positively related to coronavirus knowledge. This indicated a significant moderation of the relation between information seeking and coronavirus knowledge, with higher science knowledge strengthening the relation between information seeking and coronavirus knowledge.

Furthermore, information seeking (*β* = 0.36; *p* < 0.001) and the interaction between information seeking and trust in medicine (*β* = 0.26; *p* < 0.001) were positively related to compliance with preventive measures. Trust in medicine thus moderated the relation between information seeking and compliance with preventive measures, with higher trust strengthening the relation. There was no main effect for the relation between trust in medicine and compliance with preventive measures (*β* = 0.03; *p* = 0.243).

Finally, coronavirus knowledge was positively related to compliance with preventive measures (*β* = 0.10; *p* < 0.001). The interaction between coronavirus knowledge and trust in medicine was negatively related to compliance with preventive measures (*β* = −0.26; *p* < 0.001). This means that the relation between coronavirus knowledge and compliance with preventive measures became weaker as trust in medicine increased. Due to the negative moderation, the main effects for the relation between coronavirus knowledge and compliance with preventive measures should not be interpreted individually regarding their strength or direction. Individuals with high coronavirus knowledge and high trust in medicine reported the highest compliance with preventive measures. However, even individuals with little coronavirus knowledge reported high levels of compliance with preventive measures, if their trust in medicine was high. Conversely, individuals with low trust in medicine reported less compliance with preventive measures even if their coronavirus knowledge was high.

Table 2 displays all regression coefficients for Model 1. The model explained 22% of the variance in coronavirus knowledge and 24% of the variance in compliance with preventive measures.

**Table 2:** Regression coefficients for Model 1; Trust = trust in medicine; SE = standard error

Dependent variables	Predictors	*b*	SE	*p*	*ß*
Coronavirus knowledge	Information seeking	0.04	0.01	< .001	.22
	Science knowledge	0.28	0.02	< .001	.35
	Information seeking × science knowledge	0.02	0.00	.002	.11
Compliance with preventive measures	Information seeking	0.36	0.04	< .001	.36
	Trust	0.02	0.02	.243	.03
	Information seeking × Trust	0.04	0.01	< .001	.26
	Coronavirus knowledge	0.60	0.16	< .001	.10
	Coronavirus knowledge × Trust	−0.26	0.05	< .001	−.29

### Panic behavior (Research Question 2)

In Model 2, we investigated how information seeking related to panic behavior in a pandemic crisis and to what extent this relationship depended on specific knowledge about the coronavirus, science knowledge, and trust in medicine. All coefficients regarding the relation between information seeking and coronavirus knowledge were identical to Model 1 (see above).

Information seeking (*β* = 0.31; *p* < 0.001) and trust in medicine (*β* = 0.09; *p* < 0.001) were positively related to panic behavior. Due to the negative moderation, none of the main effects can be interpreted. The interaction between information seeking and trust in medicine was negatively related to panic behavior (*β* = −0.23; *p* < 0.001). Trust in medicine thus moderated the relation between information seeking and panic behavior, with higher trust weakening the relation. Individuals with high trust in medicine and high information seeking reported engaging in little panic behavior, as did individuals with high trust in medicine and low information seeking. Individuals with low trust in medicine reported more panic behavior regardless of their information seeking.

Finally, coronavirus knowledge was negatively related to panic behavior (*β* = −0.34; *p* < 0.001). Trust in medicine did not moderate the relation between coronavirus and panic behavior (*β* = −0.02; *p* = 0.789).

Table 3 displays all regression coefficients for Model 2. The model explained 22% of the variance in coronavirus knowledge and 17% of the variance in panic behavior.

**Table 3:** Regression coefficients for Model 2. trust = trust in medicine; SE = standard error

Dependent variables	Predictors	*b*	SE	*p*	*ß*
Coronavirus knowledge	Information seeking	0.04	0.01	< .001	.22
	Science knowledge	0.28	0.02	< .001	.35
	Information seeking × science knowledge	0.02	0.00	.002	.11
Panic behavior	Information seeking	0.27	0.03	< .001	.31
	Trust	0.06	0.02	.001	.09
	Information seeking × Trust	−0.03	0.01	.001	−.23
	Coronavirus knowledge	−1.80	0.15	< .001	−.34
	Coronavirus knowledge × Trust	−0.01	0.05	.789	−.02

## Discussion

The aim of this study was to investigate how active information seeking leads to compliance with preventive measures and panic behavior in the context of the coronavirus pandemic. We tested models assuming two pathways through which information can modify behavior: a direct path from information seeking to behavior as well as an indirect path in which information seeking leads to knowledge about the coronavirus, which in turn affects behavior. General science knowledge was assumed to help in evaluating information, whereas trust in medicine was assumed to play a crucial role in whether individuals comply with preventative measures.

The results supported our assumptions. The majority of individuals complied with preventive measures. This is crucial, as not engaging in this behavior can be considered a threat to public health. In addition, the individuals in our sample reported rather low levels of panic behavior, which is important for ensuring public order. Information seeking was directly related to both types of behavior. However, this relation was moderated by trust in medicine, with higher trust increasing the relation between information seeking and compliance with preventive measures and decreasing the relation between information seeking and panic behavior. Information seeking was particularly beneficial in leading to more compliance with preventive measures among individuals with high trust in medicine. Conversely, individuals with higher trust in medicine reported less panic behavior regardless of their information seeking behavior.

Regarding the indirect relation mediated by knowledge, individuals who sought more information also knew more about the coronavirus. Information seeking was particularly effective if individuals already possessed a general understanding of science. Greater knowledge about the coronavirus was in turn associated with more compliance with preventive measures and less panic behavior. However, even individuals with low coronavirus knowledge reported high levels of compliance with preventive measures if their trust in medicine was high. Conversely, individuals with low trust in medicine reported less compliance with preventive measures even if their coronavirus knowledge was high.

Individuals’ behavior has a strong impact on epidemiological processes during pandemics (Anderson et al. 2020; Bish and Michie 2010). On the one hand, compliance with preventive measures is critical to avoid further spreading an infectious disease or to slow down its spread (Larson and Nigmatulina 2009). On the other hand, avoiding panic behavior is important to ensure public order and prevent dangerous self-medication (Mowbray 2020). Our results indicate that science knowledge (Benjamin et al. 2017; McPhetres and Zuckerman 2018) has a prophylactic effect. Science knowledge helps individuals convert information into knowledge about the coronavirus. It provides individuals with a conceptual framework to understand and interpret the information they encounter and engage in first-order scientific reasoning by evaluating and interpreting primary evidence, thus acquiring specific knowledge (Chinn and Duncan 2018). In turn, this knowledge about the coronavirus helps people comply with preventive measures and avoid what is considered panic behavior at a given point in time.

On the other hand, trust in medicine helps individuals comply with preventive measures even if they do not fully comprehend all the facts. Highly complex situations such as pandemics are overwhelming for individuals, especially when they lack knowledge and need to process a multitude of new information (Siegrist et al. 2000; Siegrist and Zingg 2014). Trust in authorities, such as medical experts, can help individuals in their decision-making, but requires engaging in reasoning about which experts or sources to trust for the topic in question (Chinn and Duncan 2018). Our findings support the importance of trust in medicine in affecting individuals’ willingness to adopt recommended behavior (Plohl and Musil 2020; Siegrist and Zingg 2014).

Our study has some limitations that include the lack of representativeness and its cross-sectional nature. Even though our sample size is rather large, it is not a representative sample. However, the recruitment of participants in the onset of the COVID-19 pandemic was crucial for our study and the thus the criterion of representativeness was omitted due to the necessity to proceed with data collection at high pace. On the one hand, collecting data on the pandemic onset was important for our study because the actual pandemic outbreak is a critical stage in which individuals’ behavior is of high importance for the continuance of a pandemic (Anderson et al. 2020; Bish and Michie 2010). On the other hand, the early stage of pandemic outbreak was characterized by insufficient knowledge about the virus and thus, the measures of knowledge and behavior have just been established and occasionally need to be corrected with the passage of time. In this kind of situation, the use of established measures was hardly possible and the measures were dynamic in nature. This also relates to participants’ rather high performance in the coronavirus knowledge test. Without a proper norm sample to compare these results to, this may indicate a rather well-informed sample or be the result of an overly simple test. A replication of our study at a later point of the pandemic process is recommended.

Furthermore, the study’s cross-sectional nature does not allow direct causal interpretation about the nature of the reported relations. Most importantly, there may be a reciprocal relation between information seeking and knowledge, with more information resulting in higher knowledge and more knowledge resulting in an even greater interest in new information (Alexander 2003). Our findings also need to be corroborated in longitudinal and experimental designs with multiple measures for all variables. In addition, there may be additional variables such as individual differences in personality affecting individual differences in behavior that could not be considered in this study. Especially extraversion, agreeableness and Dark Triad traits such as Machiavellianism were related to how well individuals followed recommendations and should be considered in replications of our findings (e.g., Aschwanden et al. 2020; Stadler et al. 2020; Nowak et al. 2020).

Finally, the trust in medicine scale focused on trust in medical doctors as representatives of the discipline of medical sciences. The scale did not explicitly refer to trust in medical science as a scientific discipline. Trust in medical science as a discipline and trust in subdisciplines such as virology might be interesting to be included in future studies investigating the role of trust in pandemic crises.

## Conclusion

Given the importance of individuals' behavior during critical stages of a viral disease outbreak like COVID-19, facilitating science knowledge and trust in medicine through education and targeted public health messaging may be of fundamental importance for bringing crises such as the current pandemic under control. Our results highlight the importance of education in general and science education in particular. Facilitating and strengthening individuals’ science knowledge by increasing the emphasis placed on it in schools and providing adults with scientific learning opportunities—even and especially right now—can help individuals make good decisions in highly complex situations like pandemic crises. Creating opportunities especially for individuals with low coronavirus knowledge and science knowledge to understand epidemiological processes in pandemic crises might crucially affect such individuals’ behavior so that it does not pose a threat to the public. Indeed, investments in education might be investments in rational behaviors that consider the evidence derived from research processes considered to be reliable and valid.

Facilitating reasoned trust in science and medicine—i.e., second-order scientific reasoning skills (Barzilai and Chinn 2018; Chinn and Duncan 2018)—can also influence individuals’ behavior in a pandemic crisis. Trust in medicine is particularly important for individuals with low science knowledge. Trust can be influenced by the way public health communication and media campaigns are performed. Populism might endanger evidence-oriented societal discourse and thus be a threat to trust (Kienhues et al. 2020), which, in turn, is crucial for compliance and avoiding panic.

Pandemic crises and associated public health measures are challenging for everybody; only with sufficient knowledge about the disease and trust in the validity and effectiveness of the recommended measures will individuals comply with the extreme measures that are necessary to control the pandemic’s spread, limit casualties, and maintain public order.

**Michael Sailer.** Department of Psychology, Ludwig-Maximilians-Universität München, Leopoldstr. 13, 80802, Munich, Germany. E-mail: Michael.Sailer@psy.lmu.de.

*Current themes of research*:

Gamified learning, simulation-based learning and digital learning.

*Most relevant publications in the field of Psychology of Education*:

Sailer, M., Homner, L. (2020). The Gamification of Learning: a Meta-analysis. *Educational Psychology Review, 32*(1), 77–112 (2020). https://doi.org/10.1007/s10648-019-09498-w 

**Matthias Stadler.** Department of Psychology, Ludwig-Maximilians-Universität München, Leopoldstr. 13, 80802, Munich, Germany.

*Current themes of research*:

Problem-solving, reasoning, and simulations.

*Most relevant publications in the field of Psychology of Education*:

Stadler, M., Becker, N., Gödker, M., Leutner, D., & Greiff, S. (2015). Complex problem solving and intelligence: A meta-analysis*. Intelligence, 53*, 92–101. https://doi.org/10.1016/j.intell.2015.09.005 

**Elouise Botes.** University of Luxembourg, Esch-Sur-Alzette, Luxembourg.

*Current themes of research*:

International posture, language learning, education.

*Most relevant publications in the field of Psychology of Education*:

Botes, E., Gottschling, J., Stadler, M., & Greiff, S. (2020). A systematic narrative review of International Posture: What is known and what still needs to be uncovered. *System*, 102,232. https://doi.org/10.1016/j.system.2020.102232 

**Frank Fischer.** Department of Psychology, Ludwig-Maximilians-Universität München, Leopoldstr. 13, 80802, Munich, Germany.

*Current themes of research*:

Scientific reasoning and argumentation, simulation-based learning.

*Most relevant publications in the field of Psychology of Education*:

Chernikova, O., Heitzmann, N., Stadler, M., Holzberger, D., Seidel, T., & Fischer, F. (2020). Simulation-Based Learning in Higher Education: A Meta-Analysis. *Review of Educational Research*. https://doi.org/10.3102/0034654320933544 

**Samuel Greiff.** University of Luxembourg, Esch-Sur-Alzette, Luxembourg.

*Current themes of research*:

Problem-solving, reasoning, educational large-scale assessment.

*Most relevant publications in the field of Psychology of Education*:

Fiore, S. M., Graesser, A., & Greiff, S. (2018). Collaborative problem-solving education for the twenty-first-century workforce. *Nature human behaviour, 2*(6), 367–369. https://doi.org/10.1038/s41562-018-0363-y 
